# A Network Pharmacology-Based Study of Potential Targets of Angelicae Pubescentis-Herba Taxilli Compound for the Treatment of Osteoarthritis

**DOI:** 10.1155/2022/4286168

**Published:** 2022-12-28

**Authors:** Jie Zhao, Fu-Chang Zhao, Gang Li

**Affiliations:** ^1^Zhangqiu District Hospital of Traditional Chinese Medicine, Jinan, China; ^2^Affiliated Hospital of Shandong University of Traditional Chinese Medicine, Jinan, China

## Abstract

**Objective:**

Using network pharmacology and molecular docking, we explored the mechanism of Angelicae Pubescentis- (AP-) Herba Taxilli (HT) in the treatment of osteoarthritis (OA).

**Methods:**

We selected Traditional Chinese Medicine Systems Pharmacology platform (TCMSP) to filtrate the practical components and targets of AP-HT. The disease targets of “osteoarthritis (OA)” were collected by GeneCards, DrugBank, TTD, OMIM, and PharmGKB databases, and the component-target interaction network was established by Cytoscape 3.9.1. Then, we set the protein-protein interaction (PPI) network by the STRING platform and visualized by Cytoscape 3.9.1. We also conducted Gene Ontology (GO) analysis and Kyoto Encyclopedia of Gene and Genome (KEGG) pathway enrichment analysis via the bioinformatics platform. Finally, we performed molecular docking using PyMOL 2.3.0 and AutoDock Vina software.

**Results:**

11 potential compounds were selected, and 1007 OA disease targets were collected. Ninety-four main targets of the AP-HT compound in the treatment of OA have been defined. PPI network demonstrated that JUN, RELA, TNF, IL6, MAPK1, TP53, AKT1, FOS, IL10, and MYC might serve as the critical targets of AP-HT for the treatment of OA. Moreover, membrane raft, membrane microdomain, cellular response to chemical stress, and cytokine receptor binding may play essential roles in the treatment of OA via GO analysis. The main functional pathways involved in these critical targets include fluid shear stress and atherosclerosis, lipid and atherosclerosis, IL-17 signaling pathway, age-range signaling pathway in diabetic composites, and TNF signaling pathway via KEGG analysis. The results of molecular docking showed that the critical ingredients of AP-HT had an excellent affinity to related nuclear genes.

## 1. Introduction

Osteoarthritis (OA), also called degenerative osteoarthritis, degenerative arthritis, senile arthritis, and hypertrophic arthritis, is a degenerative joint disease, mostly occurring in the elderly, and is bound up with many factors such as age, weight, gender, and trauma [1]. The clinical manifestations of OA mainly include chronic joint pain, joint tenderness, morning stiffness, swelling, and common deformities. Worldwide, an estimated 240 million individuals suffer from symptomatic, activity-limiting OA [2, 3]. OA often occurs in the joints with heavy negative loads and frequent activities, such as the hip and knee joints. Research shows that 30% of people over 45 years old have imaging evidence of knee osteoarthritis, and about half have knee symptoms [4, 5]. The prevalence of symptomatic hip osteoarthritis is about 10% [6, 7]. The pathological features of OA are articular cartilage deformation, subchondral bone sclerosis and cystic change, and marginal osteophyte formation.

The main methods of OA treatment are regular exercise, proper weight loss, and education about local or oral NSAIDs among patients without contraindications. An intra-articular steroid injection can relieve pain in a short time, such as duloxetine [8]. In the late stage of OA, knee or hip replacement is often required, bringing a substantial economic burden to patients and their families. It is also worth considering that the replacement joint has a specific service life. Therefore, conservative treatment of arthritis is of great significance to patients and their families. Angelicae Pubescentis- (AP-) Herba Taxilli (HT) is a classic drug pair in the prescription of Duhuo-Jisheng decoction, which is widely used in treating OA. Angelicae Pubescentis is an Umbelliferae plant of Angelica bidentate's dry root. Its companion drug Herba Taxilli is Trichoderma's dry branch and leaf. Modern medical research shows that traditional Chinese medicine (TCM) has unique advantages and broad prospects in treating OA, but its principle needs to be further revealed and verified.

The material basis of efficacy and molecular mechanism of the above two drugs still need to be fully understood. Therefore, this study is aimed at analyzing the active components, targets, and possible approaches of AP-HT treating OA.

**Figure 1:**
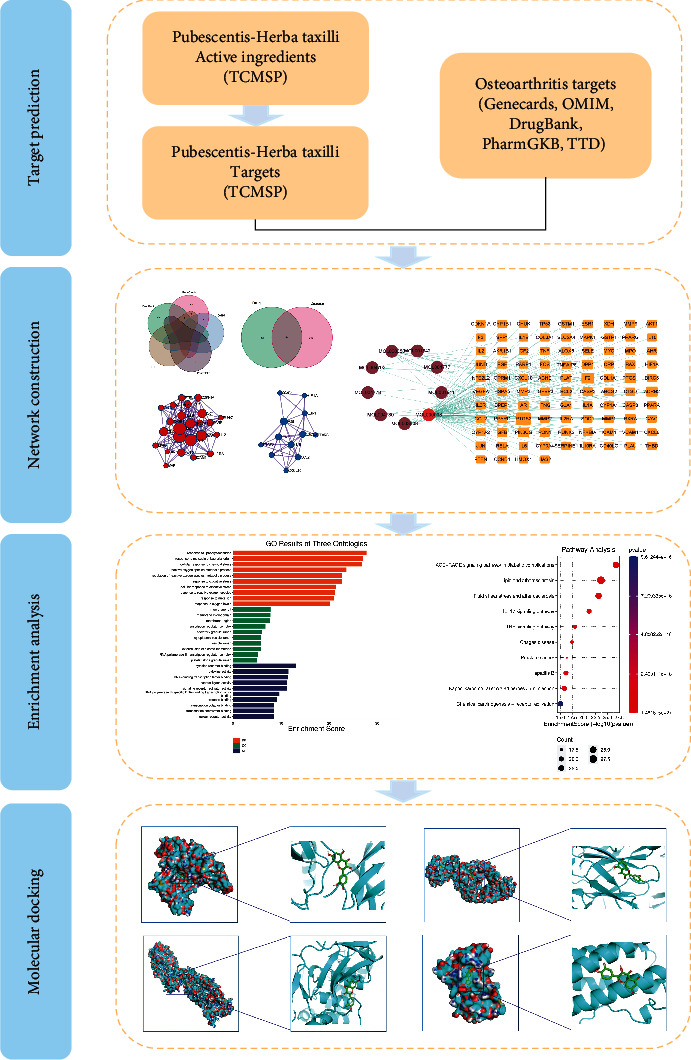
Flowchart of network pharmacology and molecular docking.

Network pharmacology is a kind of technology and knowledge involving bioinformatics, multidirectional pharmacology, computer science, and other disciplines [9]. It can explain the potential mechanism of drug treatment in multiple directions and reflect the integrity and systematization of drug action. The overall concept and treatment based on syndrome differentiation are the most significant characteristics of TCM and the difficulties in the past research of TCM. The integrity and systematic characteristics of network pharmacology can solve this problem well. They are widely used in the research of TCM [10]. Given this, we used network pharmacology to screen the practical components of AP-HT from the network database, match the targets of the functional elements with the disease targets of OA, and screen the intersection targets of drug effects. Bioinformatics analysis was carried out on the above intersection targets. A “drug component-disease target” network and protein-protein interaction (PPI) network were visualized, respectively (Figure 1).

## 2. Materials and Methods

### 2.1. Target Screening and Prediction of AP-HT and Osteoarthritis

TCMSP contains 499 herbal TCM and 12144 components of TCM, a shared database for studying TCM network pharmacology. The compounds stemming from AP-HT were assembled from TCMSP. Use the indexes of absorption, distribution, metabolism, and excretion (ADME) to screen the blood entering active components of AP-HT. To obtain compounds for further analysis, we screened the candidate compounds that met the following parameters, including oral bioavailability (OB) ≥ 30% and drug − likeness (DL) ≥ 0.18.

Through the component's target prediction function in TCMSP, the targets of AP-HT components in the blood were collected. For the convenience and accuracy of data expression, the “protein name” of the targets was converted into “gene name” in the UniProt database.

This study collected relevant disease target data using GeneCards, DrugBank, TTD, OMIM, and PharmGKB databases. Use “OA” or “osteoarthritis” keywords to search relevant disease genes. Eliminate targets with correlation values below 1 in the GeneCards database to improve data reliability. Map the drug targets with the disease targets, display the results in a Wayne diagram, and then preliminarily obtain the intersection targets of AP-HT and osteoarthritis, that is, the active target of AP-HT in the treatment of OA.

### 2.2. Construct and Analyze the Network of Compound-Target-Disease

The Cytoscape software, as an optical network construction and analysis software, is a typical software for the visual analysis of traditional Chinese medicine network pharmacology. Input target data of AP-HT treating OA, active components, and osteoarthritis collected above into the Cytoscape (version 3.9.1) software. In the network, nodes represent effective ingredients of AP-HT and disease targets of OA. Edges represent the relationship between components and targets. Then, the mechanism of AP-HT treatment of OA was preliminarily discussed by constructing and analyzing the network of compound-disease targets.

### 2.3. Construct and Analyze the PPI Network of Key Targets

The STRING database is a commonly used database for constructing protein-protein interaction (PPI) networks. It contains the protein-protein interaction relationships of various methods, including calculation, experiment, and literature. The STRING online database is used to construct the interaction network between overlapping targets to understand their interactions further. When constructing the network, the setting option is “Homo sapiens” and a confidence score ≥ 0.900 (highly trusted). Moreover, export the network diagram as a table and pictures, respectively. Construct and analyze the network by Cytoscape (version 3.9.1).

### 2.4. GO Analysis and KEGG Enrichment Analysis of Key Targets

Use the bioinformatics platform to perform Gene Ontology (GO) and Kyoto Encyclopedia of Genes and Genomes (KEGG) pathway enrichment analyses to analyze the critical target mechanism of AP-HT in the treatment of OA. When species select “human,” the *p* value is defined as < 0.05 and the *q* value as < 0.05.

### 2.5. Docking and Verification of Potential Active Ingredients

We selected molecular docking to verify the correlation of compounds in pharmacological network research. Its simulation is also applicable to promising targets. Select the PubChem database to download the 2D deconstruction of compounds and then save it as a file in mol2 format. The 3D structure of the OA docking targets (top 5 degrees in the PPI network) treated by AP-HT was downloaded from the Worldwide Protein Data Bank (PDB) database. They were imported to AutoDock Tools for hydrogenation, dehydration, and other pretreatments of dehydration. Then, the receptor molecules were docked, and the ligand was analyzed for its binding activity. The commonly used molecular image software PyMOL was used to visualize molecular docking results.

## 3. Results

### 3.1. Screening of Active Ingredients and Targets

By searching the TCMSP database, 99 different active ingredients of AP and 46 of HT were screened. After screening the conditions (OB and DL), 11 active compounds were included, including 9 compounds from AP (81.82%) and 2 compounds from HT (18.18%) (Supplementary Table 1).

**Figure 2:**
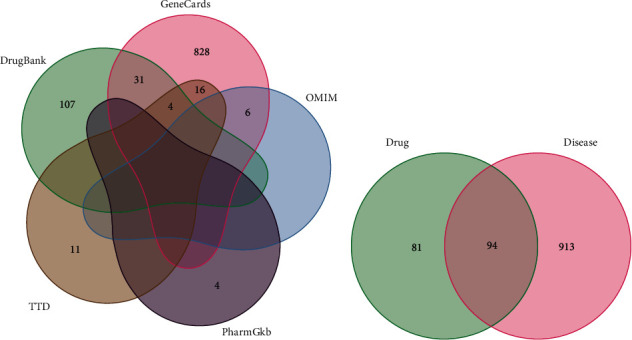
The Wayne diagram of disease target and drug targets.

Excluding the intersecting targets in the database, 1007 OA disease targets were collected, including 885 targets in the GeneCards database, six in the OMIM database, 142 in the DrugBank database, 11 targets in the TTD database, and four targets in the PharmGKB database. See Figure 2(a) for the Wayne diagram of disease targets.

A total of 175 targets of active components of AP-HT and 1007 targets of OA disease were collected. See Figure 2(b) for the Wayne diagram of overlapping targets of drugs and disease. It can be seen that there are 94 overlapping targets, which are the potential targets for the treatment of OA by AP-HT.

### 3.2. Network of Compound-Target-Disease Construction

**Figure 3:**
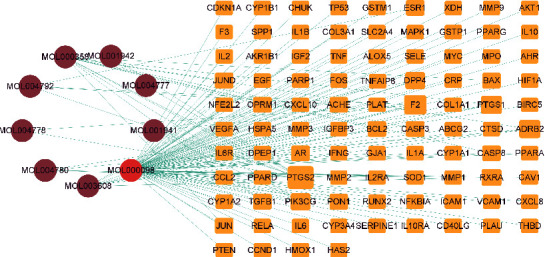
The network of compound-target-disease construction. The targets were described as yellow rectangles. The larger the node degree value, the larger the rectangle. Compounds were represented as circular. The brown circle represents the active ingredient of AP and the red of HT. The edges were regarded as the association between the nodes.

The network of compounds targeting disease was constructed using the nine significant components and 94 core targets (Figure 3). It can be seen from Figure 3 that in this network, there are eight active ingredient nodes of AP and one active ingredient node of HT (among the 11 active ingredients of drugs in Supplementary Table 1, [(1R, 2R)-2,3-dihydroxy-1-(7-methoxy-2-oxochrome-6-yl)-3-methylbutyl] 3-methyl butanoate and sitosterol have no corresponding target of OA). There are 94 potential action targets of AP-HT in the treatment of OA: quercetin, beta-sitosterol, o-acetylcolumbianetin, and angelicone are essential active ingredients.

### 3.3. PPI Network Construction

**Figure 4:**
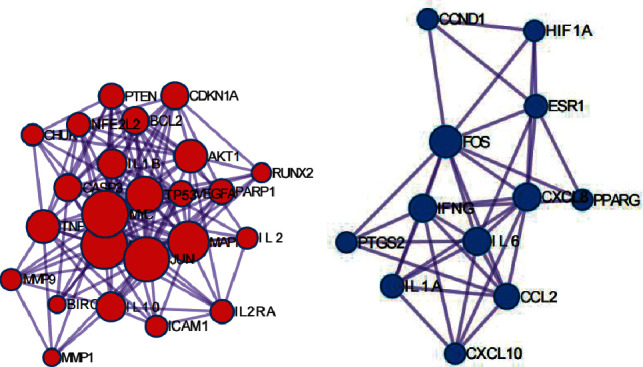
Model in PPI network of the target of Angelica pubescens and mulberry parasite for OA treatment. (a) MCODE1. (b) MCODE2.

The PPI network is constructed and analyzed to further explore the interaction between overlapping targets. The intersection targets were imported into the STRING database to construct a PPI network. Set the PPI reliability to 0.9 (highly reliable) and then output the network information. The PPI network is constructed by using Cytoscape (version 3.9.1) (Figure 4). The larger the node, the higher the degree value. That is, the more critical it is in the network. The main parameters of the network contained the number of nodes (84) and edges (306). The node degrees of proteins in the top 10 were JUN, RELA, TNF, IL6, MAPK1, TP53, AKT1, FOS, IL10, and MYC. The results indicated that the pharmacological effects of AP-HT on OA were associated with the interaction of these core proteins.

Some areas with high density in PPI complex networks are called communities or modules. The network inside the module is the potential subnet of the PPI network. The subnet connection density is high, and the regional part has few connections. Therefore, the module is considered to be a collection of biological significance. In order to more accurately analyze the mechanism of action of AP-HT in the treatment of OA, it is necessary to further identify its intrinsic modules after obtaining the PPI network of AP-HT in the treatment of OA (Figures 4(a) and 4(b)).

### 3.4. GO Enrichment Analysis

**Figure 5:**
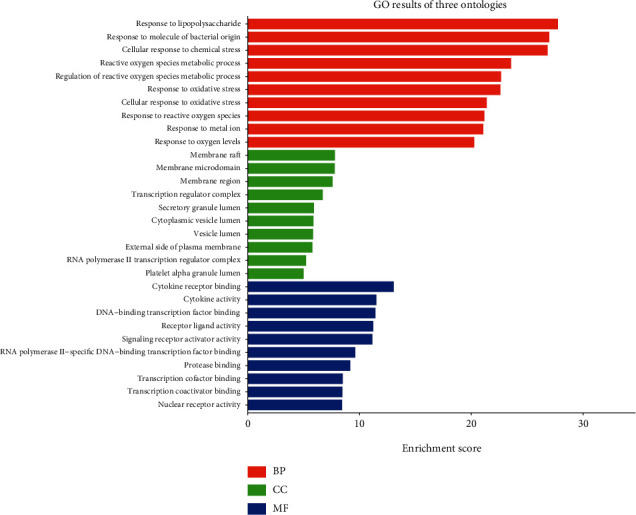
GO enrichment analysis of main component targets in AP-HT.

The molecular function GO enrichment analysis was performed on the expected action targets. Four 4161 GO items were obtained, including 3642 BP, 199 CC, and 320 MF. The results showed that AP-HT treatment of OA mainly involved BP, CC, and MF, three aspects of mediation. BP mainly includes response to lipopolysaccharide, response to molecule of bacterial origin, cellular response to chemical stress, reactive oxygen species metabolic process, regulation of reactive oxygen species metabolic process, response to oxidative stress, cellular response to oxidative stress, response to reactive oxygen species, response to the metal ion, and response to oxygen levels. CC (cellular component) is mainly enriched in “membrane raft,” “membrane microdomain,” “transcription regulator complex,” and “RNA polymerase II transcription regulator complex.” MF (molecular function) is mainly enriched in cytokine receptor binding, cytokine activity, receptor live activity, signaling receptor activator activity, and others (Figure 5).

### 3.5. KEGG Enrichment Analysis

**Figure 6:**
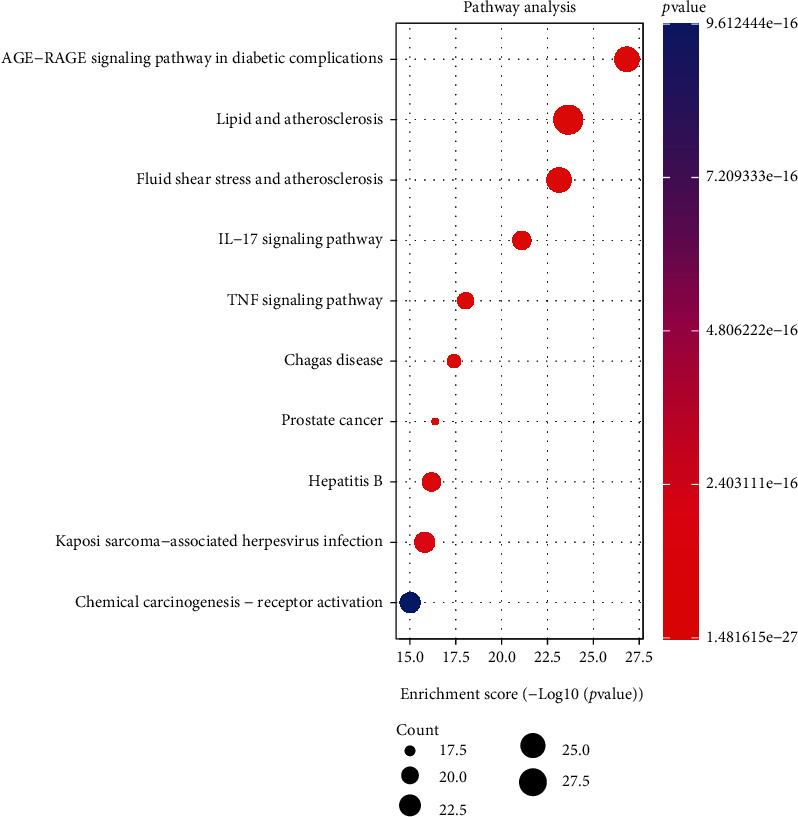
KEGG enrichment analysis of main component targets in AP-HT.

KEGG biological pathway enrichment analysis was carried out for the overlapping action targets. KEGG pathway analysis of the core targets enriched two hundred seventeen signal pathways. See Figure 6 for the top 10 biological pathways. Among them, essential pathways include the AGE-RAGE signaling pathway in diabetic complications, lipid and atherosclerosis, fluid shear stress and atherosclerosis, IL-17 signaling pathway, TNF signaling pathway, Chagas' disease, prostate cancer, hepatitis B, Kaposi's sarcoma-associated herpesvirus infection, and chemical carcinogenesis-receptor activation (Figure 6 and Supplementary Figure 1).

### 3.6. Molecular Docking Results and Analysis

**Figure 7:**
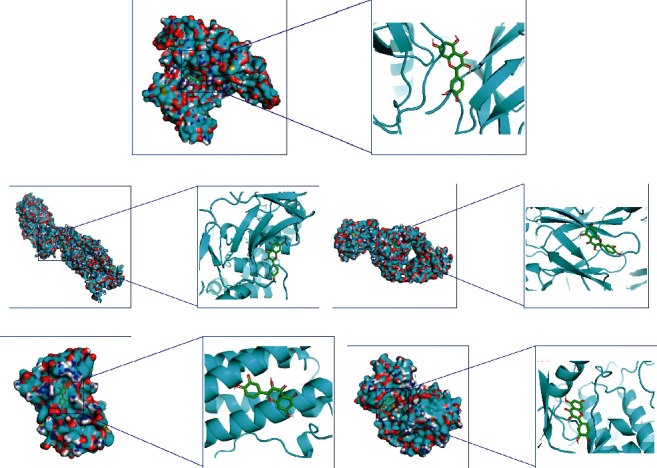
Molecular docking diagram of chemical composition to target: (a) JUN-quercetin; (b) RELA-quercetin; (c) TNF-quercetin; (d) IL6-quercetin; (e) MAPK1-quercetin.

The top 5 targets of the degree are JUN, RELA, TNF, IL6, and MAPK1. The docking targets with active components with the highest degree of quercetin of AP-HT were docking (Figure 7). As shown in Supplementary Table 2, the binding energy was all less than -5.0 kcal·mol^−1^, showing reasonable binding force.

## 4. Discussion

Some data show that OA affects about 300 million people every year and could affect many joints of the whole body. The commonly involved joints involve the knee joint, hip joint, spine joint, and small joints of the hand [11]. OA has a significant impact on individual patients, causing pain and disability. It is also an aggravating social and medical burden. Because the pathogenesis of OA is very complex, so far, there is still a lack of clinical methods to cure OA or completely prevent the development of OA disease. Globally, prevalent cases of OA increased by 113.25%, from 247.51 million in 1990 to 527.81 million in 2019. Age-standardized prevalence rates (ASR) were 6,173.38 per 100,000 in 1990 and 6,348.25 per 100,000 in 2019, with an average annual increase of 0.12% (95% confidence interval (95% CI) 0.11%, 0.14%) [12].

Duhuo-Jisheng decoction is a traditional recipe for treating osteoarthritis. Cartilage erosion is an essential indicator of OA. Studies by Liu et al. show that Duhuo-Jisheng decoction can participate in SDF-1/CXCR4/NF-*κ*B pathway. These signal pathways can effectively inhibit the production of proinflammatory cytokines and reduce the loss of type II collagen so Duhuo-Jisheng decoction can slow down the joint disease process [13]. The research of Wu et al. revealed that Duhuo-Jisheng decoction could significantly promote DNA synthesis in the proliferation cycle of chondrocytes and raise the expression of cyclin D1, CDK4, CDK6, and Rb. The above conclusions indicate that Duhuo-Jisheng decoction can promote the proliferation of articular chondrocytes, which is crucial to the prevention and treatment of OA [14]. Zhang et al. objectively and systematically evaluated the papers on treating knee osteoarthritis with Duhuo-Jisheng decoction combined with western medicine in the online database before October 12, 2015. The results showed that Duhuo-Jisheng decoction could significantly reduce the affected part's pain and improve the affected limb's function when combined with glucosamine, meloxicam, or hyaluronic acid injection for more than four weeks [15].

AP-HT is the central drug pair in Duhuo-Jisheng decoction and has specific therapeutic effects on OA. The internal regulation of disease by the body is a very complex process. A single target or a single signal pathway cannot play a therapeutic role. Different targets and signal pathways must be related and cooperate to play a role. The results of TCMSP database retrieval and “component target” network analysis in this study showed that quercetin, beta-sitosterol, o-acetylcolumbianetin, angelicone, quercetin, *β*-sitosterol, acetate, and Angelica sinensis ketone are the main practical components of AP-HT drug pair. The research conducted by Hu et al. showed that quercetin could not only increase osteoblast differentiation but also inhibit osteoclast bone resorption and induce its apoptosis to maintain the balance of bone metabolism and prevent the further development of OA [16]. Quercetin can stimulate the production of normal peripheral blood mononuclear cell (PBMC) derivatives and can also inhibit COX-2, nuclear factor-*κ*B (NF-*κ*B), activator protein-1 (AP-1), mitogen-activated protein kinase (MAPK), reactive nitric oxide synthase (NOS), and inflammatory C-reactive protein (CRP). A study conducted by Li et al. has shown that quercetin can improve cartilage repair under an OA environment by inhibiting inflammation and apoptosis of chondrocytes, regulating the polarization of synovial macrophages to M2 macrophages, and creating a chondrogenic environment for chondrocytes, thus playing a role in cartilage protection [17]. Through the above ways, quercetin can effectively regulate the inflammatory response in vivo. Qian et al.'s research showed that *β*-sitosterol could inhibit synovial angiogenesis by affecting the proliferation and migration of endothelial cells, thus reducing joint swelling and cartilage damage in CIA mice. It can inhibit the expression of the VEGF signaling pathway, slow joint inflammation, and improve the damage of bone and cartilage [18].

The PPI network analysis results of AP-HT in the treatment of OA showed that among the 94 target proteins, JUN, RELA, TNF, IL6, MAPK1, TP53, AKT1, FOS, IL10, and MYC ranked in the top of the node degree value, which may be the critical targets for AP-HT to exert the therapeutic effect. Studies by Cen et al. showed that CXCL8 might play an essential role in the migration of CD4+ T cells mediated by bone marrow mesenchymal stem cells (MSCs), and the autophagy of MSCs is positively correlated with the expression of CXCL8 mRNA, thus participating in the inflammatory response [19]. IL6 is an inflammation-related factor. Its expression plays a crucial role in secretory cells related to the differentiation of lymphocytes and monocytes and is an essential link in the progression of OA. EGF is a tyrosine kinase receptor that plays a vital role in maintaining superficial chondrocytes during articular cartilage development. It is one of the receptors for cell proliferation and signal transduction. Its activation can mediate the biological processes, such as the growth, proliferation, and differentiation of osteoclasts in arthritis [20]. Raghu et al.'s test data revealed that the decreasing number of monocytes/macrophages in the joints of mice was consistent with lacking CCL2, the result of which effectively prevented the progression of OA in mice. At the same time, their studies showed that the level of CCL2 in the synovial fluid of OA patients was significantly higher than that of ordinary people (CCL2/CCR2, but not CCL5/CCR5, mediates monocyte recruitment, information, and utilization destruction in osteoarthritis) [21].

AKT1 is a serine/threonine protein kinase. Studies have shown that AKT1 is related to cell apoptosis, and inhibiting the expression of AKT1 can promote chondrocyte apoptosis [22]. HMOX1 can promote adipogenic and osteogenic differentiation of MSCs under the induction of bone morphogenetic protein 9 (BMP9) and may enhance osteogenesis by regulating multiple signaling pathways [23].

The results of the KEGG enrichment analysis showed that AP-HT could treat OA through multiple signal pathways. Potential targets are mainly enriched in the AGE-RAGE signaling pathway in diabetes complications, lipids and atherosclerosis, fluid shear stress and atherosclerosis, IL-17 signaling pathway, TNF signaling pathway, and others. They mainly involve complex biological processes such as cell differentiation, apoptosis, metabolism, and inflammatory response. Cell differentiation and apoptosis include the TNF signaling pathway, MAPK signaling pathway, HIF-1 signaling pathway, and C-type lectin receptor signaling pathway. The HIF-1 signaling pathway is a hypoxia signaling pathway, which can effectively enhance the osteogenic and angiogenic differentiation ability of bone marrow mesenchymal stem cells. MAPK regulates cell proliferation and differentiation and plays a vital role in maintaining bone metabolic balance. Metabolic-related signaling pathways include AGE-RAGE signaling pathways in diabetic complications, rheumatoid arthritis, and endocrine resistance. *Rheumatoid arthritis* is an inflammatory disease that invades the joint synovium. Inflammatory factors and proliferative synovium tissue destroy the articular cartilage and subchondral bone, resulting in enhanced osteoclast activity, thus accelerating the process of bone resorption. Inflammatory reactions include human cytomegalovirus infection, hepatitis B, and salmon infection. In the process of disease development of OA, inflammatory factors participate in cartilage erosion and accelerate the development of OA [24].

## 5. Conclusions

To sum up, the potential practical components of AP-HT for the treatment of OA mainly include quercetin, beta-sitosterol, o-acetylcolumbianetin, angelicone, and others. These components above can play a therapeutic role in OA by directly or indirectly participating in cell differentiation, apoptosis, metabolism, and inflammatory response and have the characteristics of multicomponent, multitarget, and multisystem. Based on the network pharmacology analysis, we can obtain the possible practical components and potential action targets of AP-HT in the treatment of OA. However, this study is only a prediction analysis, and the above conclusions and specific mechanisms of action need to be demonstrated in subsequent studies.

## Data Availability

The data used to support the findings of this study are available from the corresponding author upon request.

## Supplementary Materials

Supplementary 1 Supplementary Table 1: chemical information sheet of major active ingredients.

Supplementary 2 Supplementary Table 2: top 10 targets of PPI.

Supplementary 3 Supplementary Figure 1: important targets and pathways of KEGG.
