# Supplementary material for: A Network Pharmacology-Based Study of Potential Targets of Angelicae Pubescentis-Herba Taxilli Compound for the Treatment of Osteoarthritis
Source: Comput Math Methods Med. 2022 Dec 28;2022:4286168. doi: 10.1155/2022/4286168 (PMC9814887; doi:10.1155/2022/4286168)
Supplement: Supplementary 1 — Supplementary Table 1: chemical information sheet of major active ingredients. [file 4286168.f1.docx]

Supplementary. TABLE I

Chemical information sheet of major active ingredients

| MOL | ingredients | OB(%) | DL | Medicine |
| --- | --- | --- | --- | --- |
| MOL001941 | Ammidin | 34.55 | 0.22 | Angelicae Pubescentis |
| MOL001942 | isoimperatorin | 45.46 | 0.23 | Angelicae Pubescentis |
| MOL000358 | beta-sitosterol | 36.91 | 0.75 | Angelicae Pubescentis |
| MOL003608 | O-Acetylcolumbianetin | 60.04 | 0.26 | Angelicae Pubescentis |
| MOL004777 | Angelol D | 34.85 | 0.34 | Angelicae Pubescentis |
| MOL004778 | [(1R,2R)-2,3-dihydroxy-1-(7-methoxy-2-oxochromen-6-yl)-3-methylbutyl] (Z)-2-methylbut-2-enoate | 46.03 | 0.34 | Angelicae Pubescentis |
| MOL004780 | Angelicone | 30.99 | 0.19 | Angelicae Pubescentis |
| MOL004782 | [(1R,2R)-2,3-dihydroxy-1-(7-methoxy-2-oxochromen-6-yl)-3-methylbutyl] 3-methylbutanoate | 45.19 | 0.34 | Angelicae Pubescentis |
| MOL004792 | nodakenin | 57.12 | 0.69 | Angelicae Pubescentis |
| MOL000359 | sitosterol | 36.91 | 0.75 | Herba taxilli |
| MOL000098 | quercetin | 46.43 | 0.28 | Herba taxilli |
