# Supplementary material for: A Network Pharmacology-Based Study of Potential Targets of Angelicae Pubescentis-Herba Taxilli Compound for the Treatment of Osteoarthritis
Source: Comput Math Methods Med. 2022 Dec 28;2022:4286168. doi: 10.1155/2022/4286168 (PMC9814887; doi:10.1155/2022/4286168)
Supplement: Supplementary 2 — Supplementary Table 2: top 10 targets of PPI. [file 4286168.f2.docx]

Supplementary. TABLE 2

Top 10 targets of PPI

| Core target | Entry | PDB ID | Binding  energy (kcal/mol) |
| --- | --- | --- | --- |
| JUN | P05412 | 1A02 | -8.2 |
| RELA | Q04206 | 7LEU | -7.7 |
| TNF | P01375 | 7KPA | -7.8 |
| IL6 | P05231 | 1ALU | -6.8 |
| MAPK1 | P28482 | 1PME | -8.5 |
