# Supplementary figures and images for: A Network Pharmacology-Based Study of Potential Targets of Angelicae Pubescentis-Herba Taxilli Compound for the Treatment of Osteoarthritis
Source: Comput Math Methods Med. 2022 Dec 28;2022:4286168. doi: 10.1155/2022/4286168 (PMC9814887; doi:10.1155/2022/4286168)

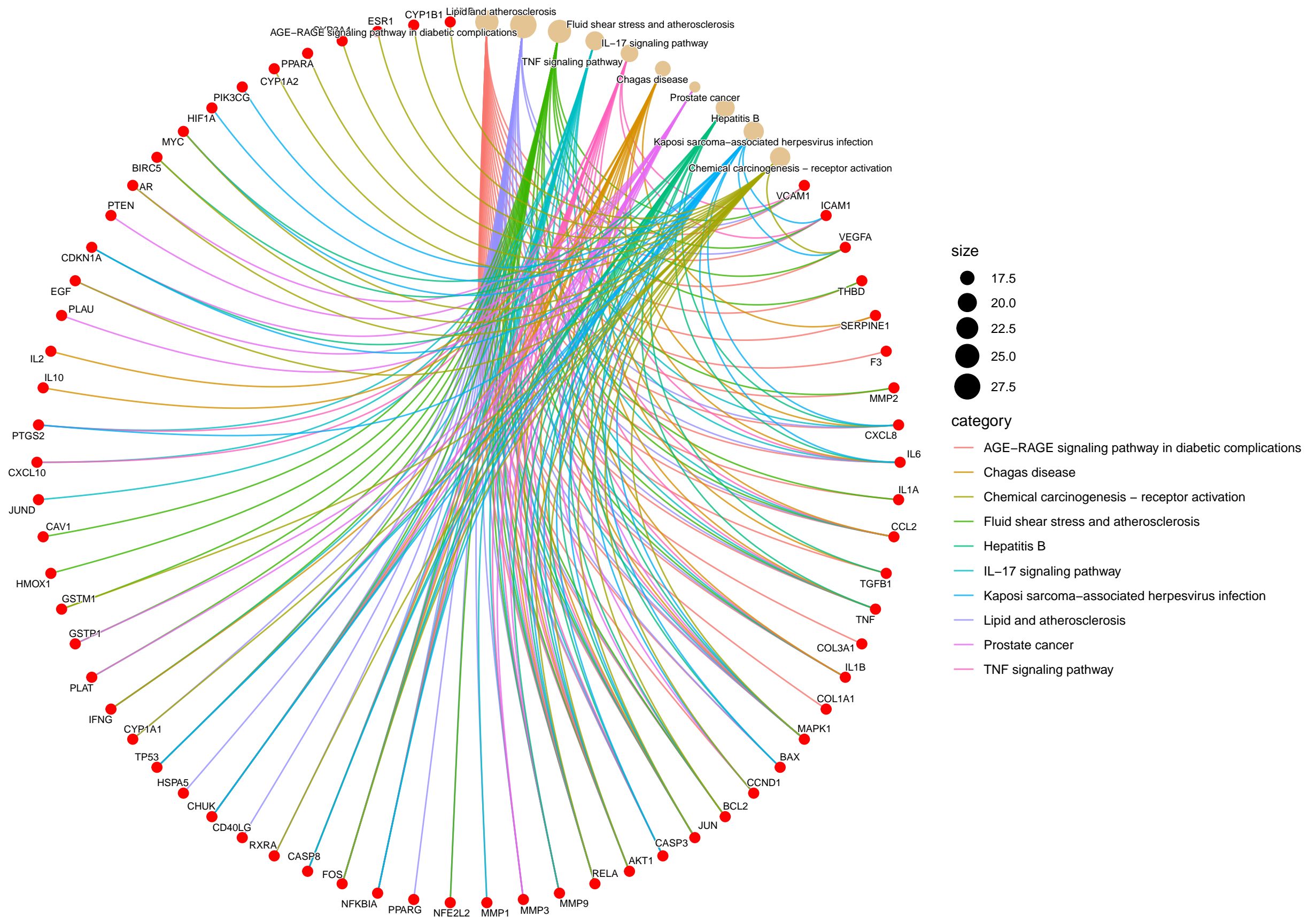

Supplement: Supplementary 3 — Supplementary Figure 1: important targets and pathways of KEGG. [file 4286168.f3.pdf]
